# Retinal Pigment Epithelium Cell Line ARPE-19 Exposed to M1 Microglia Releases Proinflammatory Cytokines and Reactive Oxygen Species Through MAP-Kinase Pathway

**DOI:** 10.3390/brainsci16060568

**Published:** 2026-05-28

**Authors:** Michela Pizzoferrato, Benedetto Falsini, Giuseppe Tringali, Pierluigi Navarra, Lucia Lisi

**Affiliations:** 1 Pharmacology Section, Department of Translational Medicine and Surgery, Università Cattolica del Sacro Cuore, Largo F. Vito 1, 00168 Rome, Italy; michela.pizzoferrato@gmail.com (M.P.); giuseppe.tringali@unicatt.it (G.T.); lucia.lisi@unicatt.it (L.L.); 2 Macula & Genoma Foundation, 00133 Rome, Italy; bfalsini@gmail.com; 3 Scientific Commettee, Università Cattolica del Sacro Cuore, Largo F. Vito 1, 00168 Rome, Italy

**Keywords:** retinal pigment epithelium (RPE), oxidative stress, MAPK signaling pathway, microglia

## Abstract

**Background**: The retinal pigment epithelium (RPE) plays a pivotal role in the visual process by maintaining the blood–retina barrier, protecting the retina from oxidative stress, and regulating immune responses. Consequently, dysfunction or degeneration of the RPE is implicated in a broad spectrum of retinal disorders that lead to progressive and irreversible vision loss. In this context, inflammation of the RPE has emerged as a critical factor in the pathogenesis of retinal degenerative diseases, underscoring its dual role as both a target and mediator of retinal inflammatory processes within the retina. **Objectives**: This study aims to preliminarily investigate, mainly by assessment of proinflammatory cytokine gene expression and immunoblotting, the molecular mechanisms underlying RPE inflammation induced by interactions between the RPE and microglia of the central nervous system. **Methods/Results**: Using in vitro models of human RPE cells, the ARPE 19 cell line was exposed to conditioned media from microglia (CHME-5 cell line) under basal and proinflammatory conditions. We observed increased activation of the MAPK signaling pathway, (evidenced by a 4-fold increase in the phosphorylation ratio of MEK and ERK) alongside elevated expression of proinflammatory cytokines, assessed by RT-PCR and immunoblotting, and a 2-fold increase in reactive oxygen species levels in RPE cells, evaluated by colorimetric assays, after exposure with conditioned media. Specifically, IL-1β and IL-8 levels increased more than 40-fold, while IL-6 expression showed a 4-fold increase compared to controls. **Conclusions**: These findings emphasize the central role of the RPE in retinal inflammation and suggest potential therapeutic targets to modulate immune responses and preserve retinal function.

## 1. Introduction

A type of specialized macrophages in the central nervous system (CNS), microglial cells play a crucial role in monitoring brain environment and initiating an inflammatory response when potential threats are detected [1]. Microglial cells can be divided into two distinct phenotypes: a proinflammatory phenotype and an anti-inflammatory phenotype. Such polarization occurs in response to different micro-environmental disturbances and plays a crucial role in regulating inflammation in the CNS, contributing both to the defense against threats and to the resolution of inflammation and restoration of homeostasis [2]. In the ocular landscape, microglia represent the resident tissue macrophages and play important roles in retinal homeostasis, recovery from injury and progression of disease [3]. In pathological conditions, activated retinal microglia interact closely with RPE (retinal pigmented epithelium), causing structural and functional changes such as reduced RPE65 and tight junction proteins, disrupted cell junctions, altered morphology, and loss of monolayer organization, which lead to irregular cellular aggregates. Microglia-derived chemokines like CCL2, CCL5, and SDF-1 further recruit immune cells and amplify inflammation [4]. In contrast, healthy RPE cells maintain an immunosuppressive subretinal environment through factors such as TGF-β, thrombospondin-1, and somatostatin, keeping microglia quiescent and the outer retina largely free of immune cells [5].

Considered as an extension of the CNS, the retina shares many similarities with the brain, in terms of structure, functions, response to injury and other immunological characteristics. Several major neurodegenerative disorders are associated with retinal manifestations, suggesting that eyes can serve as a “window into the brain’s health”. In fact, the neurodegenerative processes observed in CNS disorders are also present in various ocular pathologies, underlying common mechanisms of damage [6]. As an example of this relationship, a strong association exists between retinal neurodegeneration and amyloid β (Aβ) accumulation in Alzheimer’s disease; this association has been investigated as a possible, easily accessible biomarker for identifying individuals at high risk of developing the disease or subjects in the preclinical stages. Retinal abnormalities have been frequently reported in Parkinson’s disease, with animal models showing similar molecular mechanisms underlying Parkinson’s pathology and retinal neurodegeneration [7]. Moreover, the brain and retina share similar protective mechanisms, known as the blood–brain barrier (BBB) and blood–retina barrier (BRB) respectively, aimed at preventing the entry of unwanted cells and pathogens into the CNS or in the retina. However, when these barriers are disrupted, as in the case of injury, systemic immune cells can migrate into both the brain and retina [8]. In this regard, it is important to highlight that the integrity and functions of retinal tissues are maintained by the retinal pigment epithelium (RPE) [9]. Consisting of a single layer of polygonal cells located at the outermost part of the retina, RPE performs several essential functions, including: (a) the transport of nutrients, ions, and water, made possible by the presence of tight junctions and gap junctions; (b) the absorption of light and protection against photooxidation, by reducing the accumulation of reactive oxygen species (ROS) and oxidative damage [10]; (c) the re-isomerization of retinal for the visual cycle, phagocytosis of shed photoreceptor membranes; (d) the secretion of factors vital for retinal structural integrity; (e) the stabilization of ion composition in the subretinal space, critical for maintaining photoreceptor excitability [11].

The integrity and correct functioning of RPE cells are essential for retinal viability and homeostasis. Moreover, the interactions between RPE and the neighboring tissues, such as the choroid and photoreceptors, are crucial for maintaining the proper function of the RPE and supporting vision [12]. Unfortunately, RPE is highly vulnerable to degeneration under such conditions as age-related macular degeneration (AMD) and diabetic retinopathy [13].

In this framework, here we used human RPE and microglia cell lines to investigate the interactions between these two cell types. Incubation media obtained from human microglia taken under basal or proinflammatory-stimulated conditions were used to challenge human RPE cells, an in vitro paradigm that has been previously developed and characterized by our group to investigate the interactions between microglia and primary brain tumor cells [14].

## 2. Materials and Methods

### 2.1. Cell Cultures

ARPE-19 cells, a widely used in vitro cellular model of RPE [15], were purchased from the American Type Cell Culture (ATCC-CRL-2302, American Type Culture Collection, Manassas, VA, USA). The cells were cultured in a DMEM/F12 medium (#D6421, Sigma-Aldrich, St. Louis, MI, USA) supplemented with 10% FBS (#A5669701, Gibco; Thermo Fisher Scientific Inc., Waltham, MA, USA), L-Glutamine 2 mM (#25030081, Sigma-Aldrich, St. Louis, MO, USA), and 100 U/mL penicillin–streptomycin (#15140122, Thermo Fisher Scientific Inc., Waltham, MA, USA) at 37 °C in a 5% CO_2_ environment. The ARPE-19 cells used in this work were not polarized. The experiments were performed using cells between passage 6 and 13. Absence of Mycoplasma was confirmed before freezing via the Venor^®^ GeM Classic kit (#11-1025Minerva Biolabs, GmbH, Berlin, Germany), according to the manufacturer’s instructions.

The human immortalized brain microglia cell line (CHME-5) was kindly provided by Professor Pierre Talbot [16]. CHME-5 were cultured in DMEM High Glucose (#D6429, Corning, New York, NY, USA), containing 10% FBS (#A5669701Gibco, Thermo Fisher Scientific Inc., Waltham, MA, USA) and 100 U/mL penicillin–streptomycin (#15140122). Cells were maintained at 37 °C in a 5% CO_2_ environment and were splitted when 90% confluence was reached. All the experiments were carried out in DMEM High Glucose containing 1% FBS.

### 2.2. Chemical Reagents

All-trans-retinal (ATR) was purchased from Sigma Aldrich (#R2500, St. Louis, MI, USA). The powder was dissolved in DMSO (#D4540, Sigma Aldrich St. Louis, MI, USA), and 10 mM aliquots of stock solution were obtained. ATR working solutions were prepared fresh the day of the experiment. TNFα (#210-TA-100), IL1β (#210-LB-010) and IFNγ (#285-IF-100) were purchased from R&D system (Minneapolis, MN, USA).

### 2.3. Conditioned Media

Conditioned media from CHME-5 cells were prepared under both basal and pre-stimulated conditions as follows [17]:

Basal Conditioned Medium (M0 medium):

CHME-5 cells were plated in 25 cm^2^ flasks at a density of 70,000 cells/cm^2^. After 24 h of seeding, reaching approximately 90% confluence, CHME-5 were treated with 5 mL medium containing 1% FBS for 24 h. Thereafter, the cells were washed three times with phosphate-buffered saline (PBS, #18912014, Gibco) and fresh medium containing 1% FBS was added for a further 24 h. The resulting medium, 5 mL, was then collected, centrifuged at 1100 rpm for 5 min to remove debris, and stored at −80 °C.

Pre-Stimulated Conditioned Medium (M1 medium):

CHME-5 cells were plated in 25 cm^2^ flasks at a density of 70,000 cells/cm^2^. After 24 h of seeding, reaching approximately 90% confluence, CHME-5 were treated with 5 mL medium containing 1% FBS supplemented with a mixture of cytokines consisting of 10 ng/mL TNFα, 10 ng/mL IL-1β, and 10 IU/mL IFNγ (referred to as TII) for 24 h. Following this treatment, the cells were washed three times with PBS, and fresh medium containing 1% FBS was added. After another 24 h incubation, the 5 mL medium was collected, centrifuged at 1100 rpm for 5 min, and stored at −80 °C.

### 2.4. Viability Assays

XTT (#4891-025-K, TACS^®^ XTT Cell Proliferation Assay Kit, R&D Systems™, Minneapolis, MN, USA) was performed according to the manufacturer’s instructions, in order to choose the concentration of ATR to be used as positive control in all the experiments and to evaluate the effect of M0 and M1 media obtained from CHME-5 on ARPE-19. ATR was used in our experimental paradigm given its well-known cytotoxic properties toward RPE cells, thereby providing a reliable indication of the responsiveness of the RPE cells line [18].

In both cases, ARPE-19 were plated in a 96-well plate at a density of 7500 cells/well, reaching approximately the 80% of confluence. After 24 h from plating, the 10% FBS plating medium was replaced with 1% FBS medium containing different concentrations of ATR, ranging from 250 nM to 5 μM in the first set of experiments, and with medium M0 and medium M1 in the second set of experiments. The conditioned medium was used at different concentrations (25%, 50%, and 100%). The 25% and 50% concentrations were obtained by diluting the conditioned medium in high-glucose DMEM containing 1% FBS, whereas 100% refers to the use of the conditioned medium undiluted. This nomenclature will subsequently be used for all following experiments. In both cases, XTT assay was performed at 24 h. Cell viability was assessed by measuring absorbance at 490 nm using a microplate photometer (Victor 4, PerkinElmer, Waltham, MA, USA) and was expressed as the percentage of cell viability related to the untreated controls. All the conditioned medium dilutions were performed in DMEM High Glucose containing 1% FBS (control medium). The XTT assay measures cell viability based on the reduction of XTT by mitochondrial enzymes in living cells to form an orange formazan dye, where the color intensity correlates with the number of viable cells.

### 2.5. Bradford Assay

The Bradford assay is not a direct cytotoxicity test like the XTT assay, but it can be used in cytotoxicity studies to provide complementary information about cell number or total protein content after a treatment. Protein levels were quantified using the Bradford Protein Assay (#50000001, Quick Start™ Bradford Protein Assay Kit, Bio-Rad, Hercules, CA, USA). Cells were treated with medium M0 and medium M1, as described previously, and ATR 5 μM. After 24 h treatment, the cells were lysed in 100 μL of RIPA, admixed with a cocktail of protease inhibitor (#P8340, Sigma Aldrich) diluted 1:500. From each treatment, 10 μL of lysate was used to determine the protein concentration per well. A calibration curve was generated using bovine serum albumin, BSA (#9418 Sigma Aldrich), as a standard, with concentrations ranging from 0 mg/mL to 1 mg/mL. Protein levels were measured based on absorbance at 570 nm using a microplate photometer (Victor 4, PerkinElmer, Waltham, MA, USA), and results were expressed as total protein μg/μL per well.

### 2.6. Toxicity Assays

It is well known that the extracellular/total LDH ratio is a quantitative indicator of cell membrane integrity, therefore providing a reliable parameter for assessing cytotoxicity. Based on this evidence, the LDH assay (#G1780, CytoTox 96^®^ Non-Radioactive Cytotoxicity Assay, Promega, Madison, WI, USA) was performed in order to evaluate the cytotoxicity of our treatments on the cells. The cells were plated and treated following the same protocol used for the Bradford assay. Extracellular and intracellular LDH levels were assessed by measuring absorbance at 490 nm using a microplate photometer (Victor 4, PerkinElmer, Waltham, MA, USA) at 24 h. Extracellular LDH was quantified from cell culture medium, while intracellular LDH was determined from cell lysates. Results were expressed as the percentage of extracellular LDH relative to total LDH, where total LDH was calculated as the sum of extracellular and intracellular LDH. 

### 2.7. Western Blot

ARPE 19 cells were plated at a density of 45,000 cells/cm^2^. Experimental conditions included 24 h treatments with M0 and M1 media at 50% and 100% concentrations, as well as a separate treatment with 5 μM ATR. After 24 h treatment, cells were scraped with PBS without Ca^2+^ and Mg^2+^ and centrifuged at 1100 rpm for 5 min. Cell lysates were prepared using RIPA buffer [1 mM EDTA (#E7889), 150 mM NaCl (#S9888), 1% Igepal (#I3021), 0.5% sodium deoxycholate (#D-6750), 50 mM Tris–HCl, pH 8.0 (#T-3038), Sigma-Aldrich, St. Louis, MO, USA] with 0.1% SDS (#1610416, Bio-Rad, Hercules, CA, USA) and a protease inhibitor cocktail diluted 1:250 (#P8340, Sigma-Aldrich, St. Louis, MO, USA). Protein concentrations were determined using the Bradford protein assay as described earlier.

For electrophoresis, 50 μg of protein per sample was mixed with 4× Bolt™ LDS Sample Buffer (#B0007, Novex, Carlsbad, CA, USA) and 10× Bolt™ Sample Reducing Agent (#B0009, Novex, Carlsbad, CA, USA), boiled at 95 °C for 5 min, and loaded onto precast gels (Invitrogen, Carlsbad, CA, USA). Proteins were transferred to a PVDF membrane using the iBlot™ 2 Gel Transfer Device (Invitrogen, Carlsbad, CA, USA).

Primary antibodies were prepared in Flex Solution (iBind™ Flex Solution Kit, Invitrogen, Carlsbad, CA, USA) and incubated 2 h at room temperature or overnight at 4 °C with gentle shaking. After the incubation with the primary antibody, membranes were washed three times with TBS-T, followed by a 1 h incubation with secondary antibody in Flex Solution. After three additional TBS-T washes, protein bands were visualized using chemiluminescence (ChemiDoc™ XRS, Bio-Rad, Hercules, CA, USA) with ECL reagents (#34580 SuperSignal™ West Pico PLUS Chemiluminescent Substrate, Thermo Scientific™, Rockford, IL, USA, and Pierce™ ECL Western Blotting Substrate). Primary and secondary antibodies, and the related dilutions, are reported in Table 1.

Uncropped and unmodified Western blot images are available in the Supplementary Material.

### 2.8. Nitrite Assay

ARPE-19 cells were seeded at a density of 7500 cells/well and treated, 24 h after seeding, with medium M0 and medium M1 from CHME-5 at varying concentrations (25%, 50%, 100%) and ATR 5 μM, used as positive control. Following the 24 h treatment, the culture medium was collected to measure nitrite levels. For this, 80 μL of the medium was mixed with 40 μL of Griess Reagent (#MAK-367 Sigma-Aldrich, St. Louis, MO, USA). Absorbance was measured at 550 nm using a spectrophotometric microplate reader (PerkinElmer Inc., Waltham, MA, USA). A calibration curve (0–100 μM) was prepared using NaNO_2_ (#237213 Sigma-Aldrich, St. Louis, MO, USA) as a standard. Data were normalized based on protein quantification determined via Bradford’s method, with bovine serum albumin as the standard.

### 2.9. ROS Detection

In order to assess whether the interaction between microglia and RPE cells could induce oxidative stress phenomena, ROS levels were quantified, using the DCFDA/H2DCFDA—Cellular ROS Assay Kit (Abcam (Cambridge, UK), ab 113851). ARPE-19 cells were seeded in black multiwell plates (#3340, Corning) and treated after 24 h with the same experimental paradigm previously described. ROS levels were quantified 2 h after exposure to the treatment, using the fluorescent probe DCFDA.

Inside live cells, DCFDA is converted by esterases into a non-fluorescent form that becomes fluorescent (DCF) when oxidized by ROS.

The probe DCFDA was diluted in the kit buffer to a final concentration of 10 µM. A volume of 100 µL of the probe solution was added to each well and incubated for 45 min at 37 °C in the dark. After staining, excess dye is washed off, and new buffer is added.

Fluorescence is then measured at excitation 485 nm/emission 535 nm using a plate reader (PerkinElmer Inc., Waltham, MA, USA), whose intensity reflects the amount of ROS produced. Results are expressed as relative or fold-change fluorescence compared to Control.

Finally, to verify the accuracy of the assay, wells were included containing the kit-provided positive control, tert-butyl hydroperoxide (TBHP), at a concentration of 250 µM.

### 2.10. RNA Expression and Quantification

ARPE 19 were seeded in a 6-well plate at a concentration of 500,000 cells/well and then, 24 h after seeding, the cells were treated with medium M0 and M1 (50%, 100%) and ATR 5 μM. After 24 h-treatment, RNA was extracted using Trizol reagent (#T3809 TRI Reagent^®^, Sigma-Aldrich, St. Louis, MO, USA). The quantity of extracted RNA was determined using a Qubit fluorometer (Invitrogen, Carlsbad, CA, USA). Subsequently, 500 ng of RNA from each sample was retro-transcribed into cDNA using the PrimeScript RT Reagent Kit (#RR037A, Takara, Shiga, Japan) following the manufacturer’s instructions. Finally, the cDNA samples were diluted with nuclease-free water to achieve a final concentration of 10 μg/mL.

### 2.11. RT-PCR

Gene expression levels were quantified using real-time PCR (qPCR). The qPCR reactions were performed with the following cycling conditions: 35 cycles of denaturation at 95 °C for 20 s, followed by annealing and extension at 60 °C for 20 s. The Brilliant III Ultra-Fast SYBR Green QPCR Master Mix (#600882, Agilent Technologies, Santa Clara, CA, USA) was employed for these reactions, which were carried out in a 20 μL reaction volume using an AriaMx Real-time PCR system (Agilent Technologies, Santa Clara, CA, USA. The specific primers used for gene expression analysis are:

(a) β actin C12 F (5′-ACG TTG CTA TCC AGG CTG TGC TAT-3′) and D01 R (5′-TTA ATG TCA CGC ACG ATT TCC CGC-3′); (b) hIL1β C01 F (5′CAT GGG ATA ACG AGG CTT ATG-3′) and C02 R (5′CCA CTT GTT GCT CCA TAT CC-3′); (c) hIL6 B09 F (CCT TCC AAA GAT GGC TGA AA) and B10 R (5′-TGG CTT GTT CCT CAC TAC T); (d) hIL8 F110 (CCA GGA AGA AAC CAC CGG A) and R220 (GAA ATC AGG AAG GCT GCC AAG); (e) hCOX2 C03 F (5′ TGG CTG GCA GGG TTG CTG GTG GTA-3′) and C05 R (5′-CAT CTG CCT GCT CTG GTC AAT CGA A-3′); (f) hMCP-1 F199 (GAT CTC AGT GCA GAG GCT CG) and R351 (TGC TTG TCC AGG TGG TCC AT); (g) hTGFβ C08 F (5′-CAG TCA CCA TAG CAA CAC TC-3′) and C09 R (5′-CCT GGC CTG AAC TAC TAT CT-3′); (h) hVEGF-A F (5′-AAA TCC CTG TGG GCC TTG CT-3′) and R (5′-TTT CTG CTG TCT TGG GTG CAT TGG-3′).

### 2.12. Statistical Analyses

Each experiment was repeated at least three times. All the statistical analyses were performed with Prism™ 10.4 computer program (GraphPad, San Diego, CA, USA). Data were analyzed by one-way ANOVA, followed by Dunnett’s test and Tukey’s test. Statistical significance was determined at α = 0.05 level. Differences were considered statistically significant when *p* < 0.05.

## 3. Results

### 3.1. Effect of All-Trans-Retinal (ATR) on Cell Viability in ARPE-19

**Figure 1:**
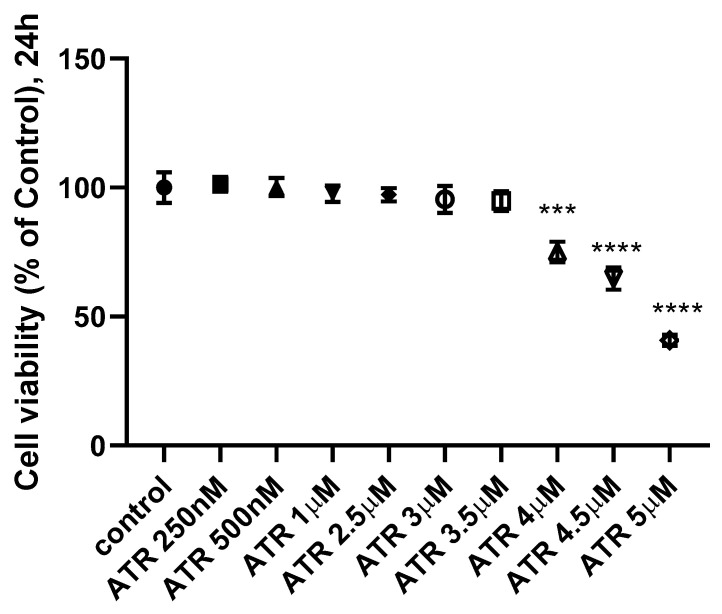
Evaluation of the effect of ATR on cell viability in ARPE 19 at 24 h. ARPE-19 cells were treated for 24 h with All-trans-retinal (ATR) at concentrations ranging from 250 nM to 5 μM to evaluate its impact on cell survival. Cell viability was determined by XTT assay and results are expressed as a percentage relative to untreated cells (Control = 100%). Data are presented as the mean ± SEM of *N* = 3 independent experiments, each performed with *n* = 6 technical replicates per experimental group. Statistical significance was assessed by one-way ANOVA followed by Dunnett’s post hoc test. *** *p* < 0.005, **** *p* < 0.0001 vs. Control.

All-trans-retinal (ATR) is a crucial intermediate in the visual cycle and its accumulation in retinal pigment epithelium (RPE) cells causes mitochondrial dysfunction and ER stress [18]. Based on these considerations, in our study we used ATR as a standard noxious stimulus on ARPE. In the first series of experiments, cells were treated with concentrations of ATR ranging from 250 nM to 5 μM [19]. Cell viability was assessed after 24 h treatment by XTT assay. As shown in Figure 1, cell viability decreases by 25% with 4 μM ATR, reaching approximately a 60% decrease after treatment with 5 μM ATR. This concentration of ATR was therefore chosen as our positive control for all the following experiments.

### 3.2. Effect of Medium M0 and M1 on Cell Viability in ARPE-19

**Figure 2:**
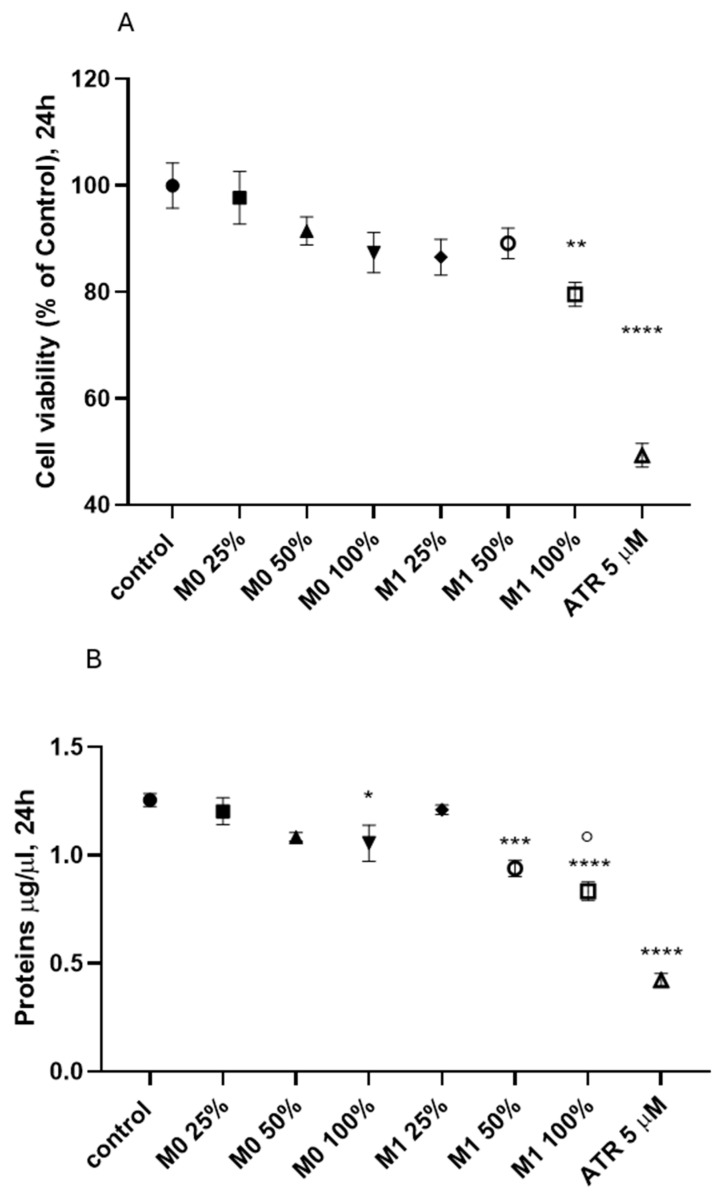

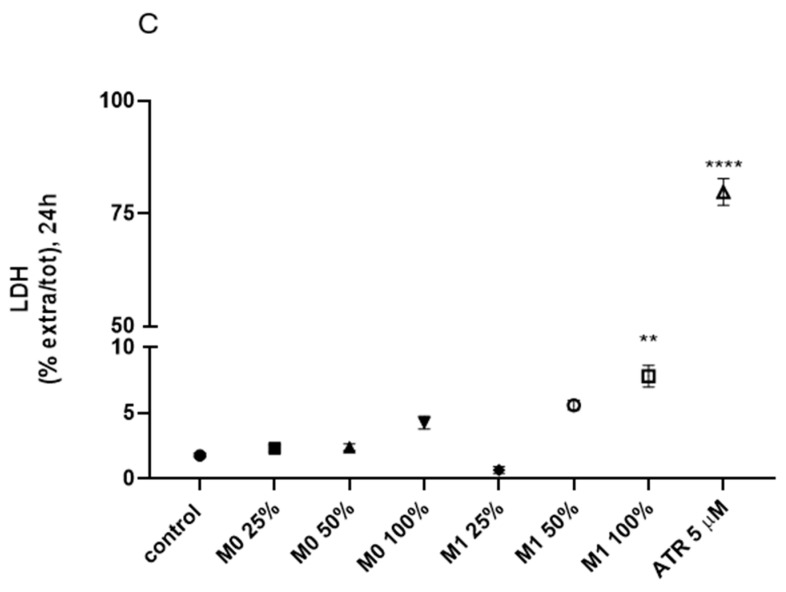
Evaluation of treatment toxicity of medium M0 and M1 medium and its effect on cell viability in ARPE 19. ARPE-19 cells were treated for 24 h with different concentrations (25%, 50%, and 100%) of M0 and M1 media. All-trans-retinal (ATR, 5 μM) was used as a positive control for cell damage. (**A**) Cell viability was assessed by XTT assay and expressed as a percentage relative to untreated cells (Control = 100%). (**B**) Total protein content was quantified to evaluate cell mass and expressed in μg/μL. (**C**) Cytotoxicity was determined by calculating the ratio of extracellular to total Lactate Dehydrogenase (LDH) activity, with results expressed as a percentage. Data are presented as mean ± SEM of *N* = 3 independent experiments, with *n* = 6 technical replicates per group in each experiment. Statistical analysis was performed using one-way ANOVA followed by Tukey’s test * *p* < 0.05, ** *p* < 0.005, *** *p* < 0.001, **** *p* < 0.0001 vs. Control; ° *p* < 0.05 M1 100% vs. M0 100%.

To mimic the interaction between microglia, both under resting M0 and proinflammatory M1 state [2] and retinal pigmented epithelium, ARPE-19 cells were exposed to conditioned media from microglia obtained under basal (M0 medium) and proinflammatory conditions (M1 medium). After 24 h of treatment with medium M0 and M1 at various concentrations (25%, 50% and 100%), we found that M0 medium has no significant effect versus Controls on both cell viability and LDH release (Figure 2A,C), while significantly reducing protein content at 100% concentration (Figure 2B). Conversely, M1 medium caused a concentration-dependent decrease in both cell viability and protein content (Figure 2A,B), and in parallel a concentration-dependent increase in LDH release (Figure 2C), with maximal effect at 100% concentration. In no case, the effects of conditioned media on these parameters reached the effect level of 5 µM ATR (Figure 2A–C). Moreover, an additional LDH assay is available in the Supplementary Material.

### 3.3. Effect of Medium M0 and M1 on Apoptosis in ARPE-19 Cells

**Figure 3:**
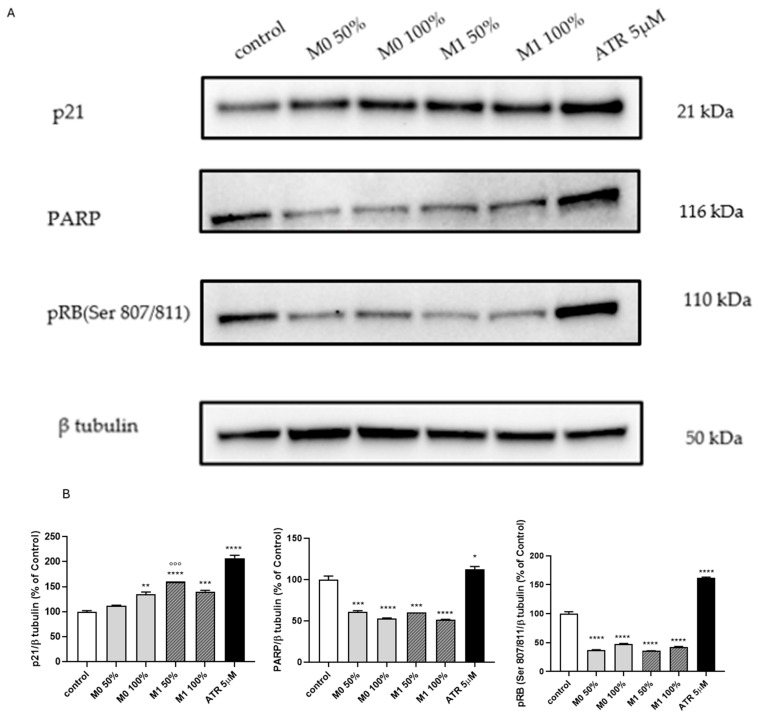
Western blot analysis of the main proteins involved in regulating apoptosis in ARPE 19 after 24 h treatments. ARPE-19 cells were treated for 24 h with M0 and M1 media (at 50% and 100% concentrations) or ATR (5 μM) as a positive control. (**A**) Representative Western blot bands showing the expression of key proteins involved in the regulation of apoptosis, such as p21, PARP and pRb (Ser 807/811); β-tubulin was used as the normalizer gene. Lane assignments: 1: Control; 2: M0 50%; 3: M0 100%; 4: M1 50%; 5: M1 100%; 6: ATR 5 μM. (**B**) Densitometric analysis of the protein bands, with values normalized to β-tubulin. Data are presented as mean ± SEM. Statistical significance was assessed via one-way ANOVA followed by Tukey’s post hoc test. * *p* < 0.05, ** *p* < 0.005, *** *p* < 0.001, **** *p* < 0.0001 vs. Control; °°° *p* < 0.001 M1 50% vs. M0 50%.

In a further series of experiments, we investigated the potential causes of the decrease in cell viability and the increase in cell toxicity observed following treatment with the conditioned medium. First, we tested the hypothesis that the microglia/retinal epithelial interaction could have effects on apoptotic mechanisms. Therefore, we evaluated in ARPE cells the effect of the conditioned M0 and M1 media on key proteins involved in the initiation of apoptosis, such as p21, PARP, and RB [20,21]. Figure 3 shows that the levels of p21 increase in a concentration-dependent manner with M0 medium, while they decrease in a concentration-independent manner with M1 medium. Regarding PARP, its expression levels decreased in a dose-dependent manner. Similarly, a significant reduction in pRb levels compared to Control was observed in all treatment groups; notably, this effect was more marked at the 50% than at the 100%.

### 3.4. ROS and NO Release

**Figure 4:**
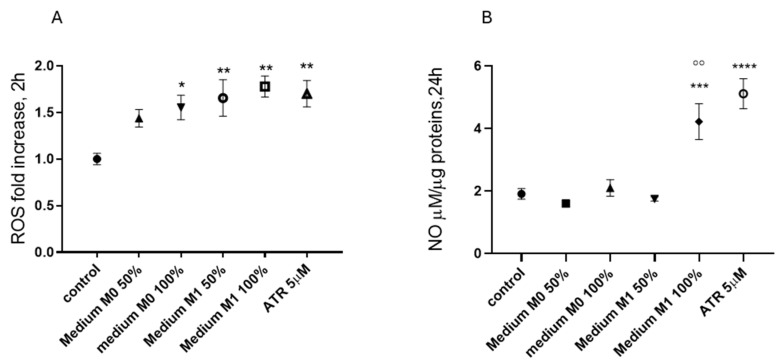
Evaluation of the effect of medium M0 and M1 on ROS (panel (**A**)) and NO (panel (**B**)) release in ARPE 19 after 24 h treatment. ARPE-19 cells were treated with M0 and M1 media for 24 h to evaluate (**A**) reactive oxygen species (ROS) production and (**B**) nitric oxide (NO) release. ATR (5 μM) was employed as a positive control for both assays. ROS levels are expressed as fold change relative to untreated cells, while NO levels are reported as μM NO/μg of total proteins. Data are presented as mean ± SEM from *N* = 3 independent experiments, each performed with *n* = 6 technical replicates per experimental group. Statistical significance was assessed by one-way ANOVA followed by Tukey’s post hoc test. * *p* < 0.05, ** *p* < 0.01, *** *p* < 0.001, **** *p* < 0.0001 vs. Control; °° *p* < 0.005 M1 100% vs. M0 100%.

Since oxidative stress and inflammation are known to negatively impact the viability of RPE cells [10,22], we assessed whether ARPE-19 produce nitric oxide (NO) and reactive oxygen species (ROS) following treatments with the conditioned media. As soon as after two hours of treatment, we observed that both the M0 and M1 conditioned media increase ROS production in a dose-dependent manner, although the increase is even more pronounced with M1 media (Figure 4A). Concerning nitric oxide, a significant and considerable increase in nitric oxide release levels after 24 h treatment was only observed after exposure to 100% M1, but not with M1 at lower concentrations or M0 at any concentration tested (Figure 4B).

### 3.5. Effect of Medium M0 and M1 on the MAPK Pathway in ARPE-19 Cells

**Figure 5:**
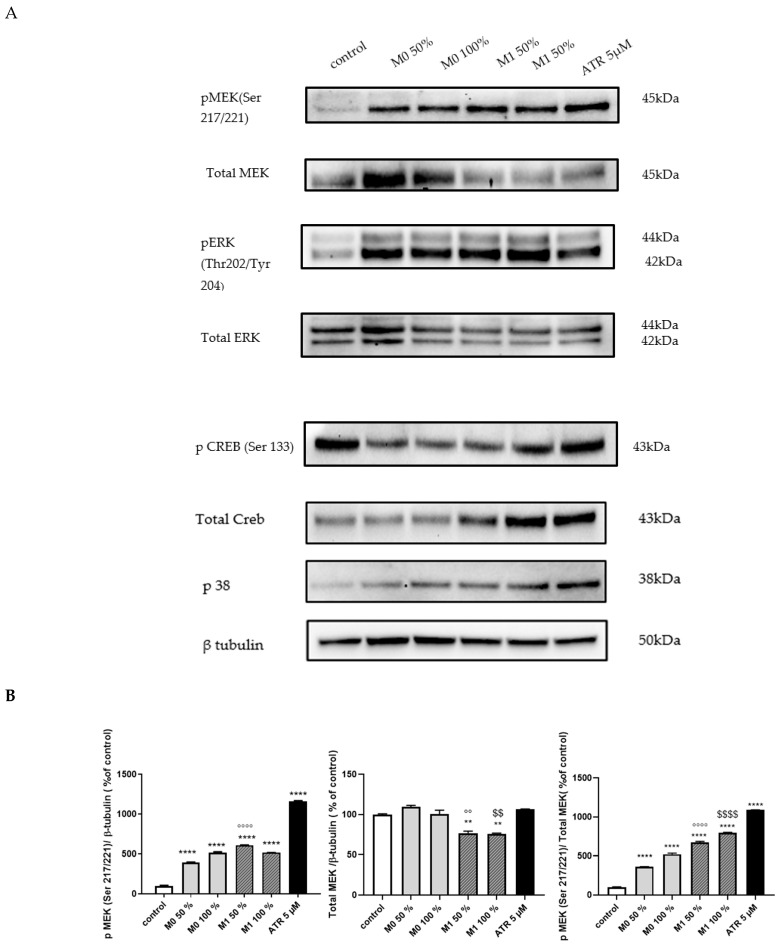

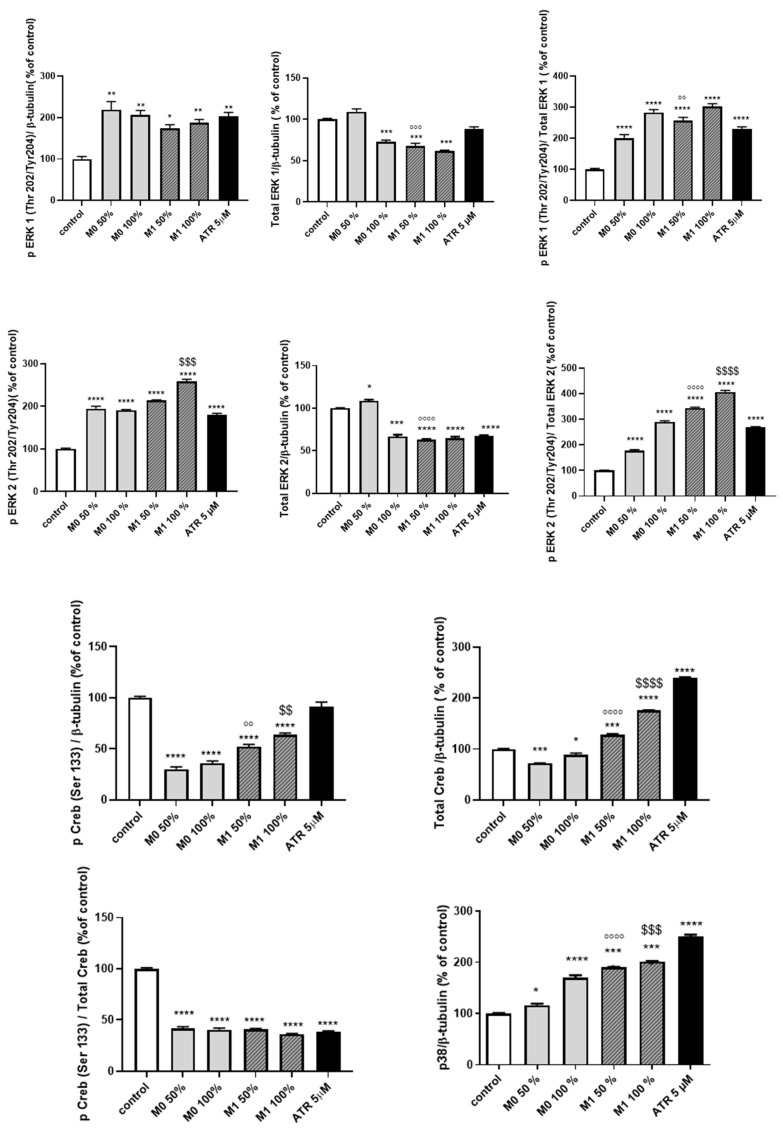
Western blot analysis of the main proteins involved in MAPK pathway in ARPE 19 after 24 h treatment. The activation of the MAPK pathway was investigated in ARPE-19 cells following a 24 h exposure to M0 and M1 media (50% and 100%) or ATR (5 μM). (**A**) Immunoblotting images display the protein bands for the targeted markers; β-tubulin served as the normalizer gene. The lanes are identified as follows: 1: Control; 2: M0 50%; 3: M0 100%; 4: M1 50%; 5: M1 100%; 6: ATR 5 μM. (**B**) Relative densitometric quantification of the bands, with values normalized against β-tubulin levels. All data are presented as mean ± SEM and were statistically analyzed using one-way ANOVA with Tukey’s post hoc test. * *p* < 0.05, ** *p* < 0.01 *** *p* < 0.001, **** *p* < 0.0001 vs. Control. °° *p* < 0.005, °°° *p* < 0.001, °°°° *p* < 0.0001 M0 50% vs. M1 50%. $$ *p* < 0.005, $$$ *p* < 0.001, $$$$ *p* < 0.0001 M0 100% vs. M1 100%.

Since inflammation and oxidative stress are known to lead to the activation of the MAP kinase pathway [23]), we assessed the activity of the main proteins involved in the MAP kinase pathway, i.e., MEK, ERK, CREB and p38 [24], after 24 h treatment, in order to further investigate the mechanisms that could underlie the generation of ROS and nitric oxide triggered by the interaction microglia/ARPE 19 (Figure 5).

Firstly, an increase in MEK and ERK phosphorylation versus untreated Controls was detected, with no significant differences between the various treatments. The total MEK levels increased, but not in a dose-dependent manner, whereas total ERK increased only with the 50% M0 medium. Considering the ratio between the phosphorylated and total forms of both MEK and ERK, we found a concentration-dependent increase, which was more pronounced with the 100% medium.

Regarding CREB, there was an overall decrease in the phosphorylated forms, with 100% M1 medium decreasing less than the other treatments. Regarding total CREB, no significant differences were observed with M0 medium, while a concentration-dependent increase was seen with M1 medium.

Finally, the level of p38 increases in a concentration-dependent manner following treatment with M0 medium at both concentrations. M1 medium also increased p38 levels, with no differences between the two concentrations.

Taken together, these results demonstrate that the interaction with microglia-derived factors (M0 and M1) triggers a robust activation of the MAPK/ERK signaling axis and p38 stress-activated pathway, coupled with a downregulation of CREB phosphorylation.

### 3.6. Evaluation of mRNA Levels of Inflammatory and Angiogenesis Proteins

**Figure 6:**
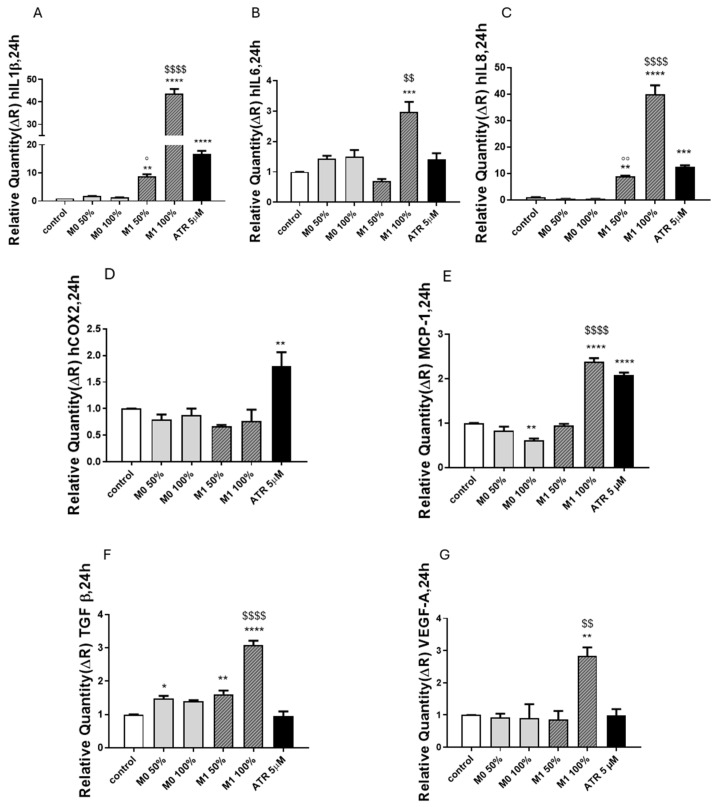
Evaluation of mRNA levels of the main proinflammatory cytokines and angiogenesis proteins. The mRNA levels of key proinflammatory cytokines and angiogenesis-related factors were evaluated by RT-qPCR after a 24 h treatment with M0 and M1 media, each tested at 50% and 100% concentrations. ATR (5 μM) was used as a positive control. Panels represent the expression levels of (**A**) hIL1β, (**B**) hIL-6, (**C**) hIL-8, (**D**) hCOX2, (**E**) hMCP-1, (**F**) hTGFβ, and (**G**) hVEGF-A. Gene expression was normalized to β-actin as the endogenous control (normalizer gene). Data are expressed as fold-change relative to untreated cells (Control), which served as the calibrator. Results are presented as the mean ± SEM of N = 2 independent experiments, each performed with n = 3 technical replicates per experimental group. Statistical analysis was conducted using one-way ANOVA followed by Dunnett’s post hoc test. Tukey’s post hoc test. * *p* < 0.05, ** *p* < 0.005, *** *p* < 0.001, **** *p* < 0.0001 vs. Control. ° *p* < 0.05, °° *p* < 0.005, $$ *p* < 0.005, $$$$ *p* < 0.0001 M0 100% vs. M1 100%.

Based on the increase in nitric oxide levels and the involvement of MAP kinases observed in the above experiments, it was also interesting to assess the gene expression of some key proinflammatory cytokines. As shown in Figure 6, we found that the expressions of IL-1β, IL-6, IL-8 and hMCP-1 are particularly elevated following the treatment with the 100% M1 medium, while M0 medium has no stimulatory effect.

The 100% M1 medium also induced an increase in TGFβ levels, which may have a role in the increased production of IL-6 and IL-8 [25]. TGFβ also induces the expression of VEGF, which is involved in the neovascularization processes underlying AMD and retinal diseases [26].

Finally, looking at COX-2, its expression is increased by the ATR, but not by incubation media.

## 4. Discussion

Considering the well-established interaction between retinal microglia and the retinal pigment epithelium [6], we sought to explore whether a similar interplay might occur between brain microglia and the RPE. One potential mechanism of interaction between microglia and RPE cells is mediated by exosomes [27]. When released from these donor microglial cells into the extracellular environment, exosomes enter a free-floating phase, circulating through systemic body fluids, enabling these microglial-derived vesicles to theoretically reach the ocular site. Current research suggests that exosomes have the capacity to modulate or even traverse the blood–retinal barrier (BRB), especially under pathological conditions where the barrier is compromised. Once they bypass this barrier, they can interact directly with RPE cells, potentially modulating their inflammatory or pathological responses [28]. The understanding of such interaction and the anatomical accessibility of the retina could offer a unique biological window for investigating cerebral pathophysiology, facilitating early diagnosis, longitudinal monitoring, and the advancement of neuroscientific research.

Inflammation of the RPE plays a crucial role in the pathogenesis of various retinal diseases, most notably age-related macular degeneration (AMD) and diabetic retinopathy [29]. The RPE, a crucial monolayer of cells supporting photoreceptor function, may undergo an inflammatory process due to various causal agents, leading to a cascade of events that may eventually contribute to vision loss [30]. RPE inflammation is triggered by various factors, primarily involving oxidative stress and the innate immune system. Moreover, our study shows that RPE can undergo inflammatory activation also upon interaction with CNS microglia. In fact, treatment of RPE cells with conditioned medium derived from CNS microglia results in a significant up-regulation of key inflammatory markers [31,32], including NO and proinflammatory cytokines such as IL-1β, IL-6, IL-8 and MCP-1. The inflammatory response is markedly enhanced when RPE cells are exposed to conditioned medium derived from microglia previously activated under M1-polarizing conditions. As it emerged from our findings, the mechanism through which this interaction may occur is likely mediated by the involvement of the mitogen-activated protein kinase (MAPK) pathway, which is known to be widely involved in inflammatory processes occurring in RPE, and in the increased production of proinflammatory cytokines [23]. Our immunoblotting data confirm that the main MAP kinases, such as MEK and ERK, are activated in the RPE primarily following treatment with M1-prestimulated conditioned medium. Nevertheless, despite a considerable increase in the phosphorylation levels of MEK and ERK (a clear indication that the pathway is activated by inflammation and ROS generation, [33]), the levels of phosphorylated CREB at serine 133, whose phosphorylation depends on the activation of several upstream factors including ERK [34], are markedly reduced. Such decrease may be explained by the ability of high ROS concentrations to lower CREB phosphorylation [35], while the initial exposure to low ROS might transiently activate CREB, sustainedly high ROS levels, as those used in our study, typically induce oxidative stress, thereby hindering CREB phosphorylation.

MAP kinases are known to play a significant role in VEGF secretion [26]; therefore, it is reasonable to hypothesize that MEK and ERK could contribute to the observed increase in VEGF-A mRNA levels. In the present project, we measured only the VEGF gene expression, however, we can speculate that a VEGF release also occurs. Translated into a human pathology setting, one such increase in VEGF-A levels may well act as one of the drivers of the pathological angiogenesis and vascular leakage characteristic of AMD, ultimately resulting in vision impairment [36]. This increase might also be attributed to elevated levels of transforming growth factor-beta (TGF-β), a cytokine known to play a key role in various cellular processes, including inflammation and angiogenesis. Indeed, TGF-β has been shown to contribute to the upregulation of VEGF expression in the retinal epithelium [26]. TGF-β may also play a role in the upregulation of IL-8 and IL-6 levels, which can synergize with IL-1β to significantly enhance both IL-6 mRNA levels and IL-6 protein production in human RPE cells [25]. Thus, under inflammatory conditions, where both cytokines are present, the effect of RPE cells on IL-6 production can be amplified, promoting the progression of inflammation and leading to a condition of overall increased cytotoxicity, an event that could be particularly driven, therefore, by the massive production of IL-1, IL-6 and IL-8. Activated microglia, particularly in the proinflammatory phenotype, release a variety of proinflammatory cytokines, which can induce oxidative stress, apoptosis, or necrosis in RPE cells. In addition, microglial activation, accompanied by the production of ROS and NO, could contribute to cellular and mitochondrial damage as well as DNA instability [37]. Furthermore, under these conditions, microglia also release lytic enzymes and proteases, which degrade the extracellular matrix and compromise the structural integrity of the RPE barrier [29], further evidence of a cytotoxic event. Within this inflammatory context, all the physiological mechanisms that normally allow microglia to remain in a quiescent state—probably mediated by the secretion of immunomodulatory factors by the RPE—may consequently be disrupted [38]. In light of this evidence, such cytotoxicity observed in the RPE could be, therefore, mediated by multiple and interrelated mechanisms.

In our in vitro model mimicking inflammation and oxidative stress, the conditioned medium—mainly the pre-stimulated one—also showed effects on apoptosis, resulting in: (a) blocking PARP activity, which makes the cell unable to repair DNA damage, thereby provoking its death [39]; (b) an increase in p21 expression levels, resulting in the inhibition of CDK kinases which in turn prevent RB phosphorylation [23].

Finally, it is important to note that the findings presented in this study are limited to in vitro experiments performed on immortalized cell lines, which unquestionably offer substantial advantages in terms of reproducibility, ease of handling, minimizing the influence of external variables and providing valuable mechanistic insights. While ARPE-19 cells are a well-established model for investigating the retinal pigment epithelium (RPE), owing to their epithelial morphology and expression of several RPE-specific genes [40], and although CHME-5 cells serve as a model for studying microglia [16,41], these models may not fully recapitulate the complexity of native cellular environments and do not fully replicate the physiological complexity of an organism. Nevertheless, this experimental model makes it possible to isolate the direct effects of a condition on a particular cell type, thereby providing valuable insights into fundamental molecular processes. Therefore, in vivo studies and generation of co-cultures will be necessary to overcome this limitation.

## 5. Conclusions 

Taken together, the present findings and the evidence from recent literature reinforce the emerging concept of a functional crosstalk between the eye and CNS, highlighting shared immunological pathways and potential bidirectional signaling mechanisms [42].

## Figures and Tables

**Table 1:** Antibodies used in the immunoblotting.

Antibody	Dilution	Producer	Catalog Number
β-tubulin III	1:1000	Sigma (St. Louis, MO, USA)	#T8578
p-MEK (Ser 217/221)	1:500	Cell Signaling (Danvers, MA, USA)	#9154
Total MEK	1:1000	Cell Signaling	#9122
p-Erk1/2 (Thr202/Tyr204)	1:1000	Cell Signaling	#9101
Total Erk 1/2	1:1000	Cell Signaling	#9102
p-CREB (Ser 133)	1:1000	ThermoFisher (Waltham, MA, USA)	#PA1-4619
Total Creb	1:500	ThermoFisher	#PA1-850
p 38	1:1000	Cell signaling	#9221
p 21	1:500	Cell signaling	#2947
p-Rb(Ser807/811) (D20B12) XP	1:1000	Cell signaling	#8516
Parp	1:1000	Cell signaling	#9532
Anti-mouse	1:3000	Sigma	#A 3682
Anti-rabbit	1:15,000	Jackson Immuno Research (West Grove, PA, USA)	#111-035-045

## Data Availability

Data are available upon request from the corresponding author. The data are not publicly available due to the big amount of raw data.

## Supplementary Materials

The following supporting information can be downloaded at: https://www.mdpi.com/article/10.3390/brainsci16060568/s1, Figure S1: Effect of antioxidant treatment with Vitamin C (200 μM and 500 μM) on toxicity induced by conditioned medium; Figure S2: Full-length, uncropped, and unprocessed scans of all Western blots presented in the main manuscript.
