# Supplementary material for: Retinal Pigment Epithelium Cell Line ARPE-19 Exposed to M1 Microglia Releases Proinflammatory Cytokines and Reactive Oxygen Species Through MAP-Kinase Pathway
Source: Brain Sci. 2026 May 28;16(6):568. doi: 10.3390/brainsci16060568 (PMC13297064; doi:10.3390/brainsci16060568)
Supplement: Supplementary file 1 [file brainsci-16-00568-s001.zip › brainsci-4295417-supplementary.pdf]

brain sciences

Supplementary Figure S1. Effect of antioxidant treatment with Vitamin C (200 μM and 500 μM) on toxicity induced by conditioned medium. Cells were exposed to conditioned medium in the presence or absence of an antioxidant. Cytotoxicity was evaluated by measuring LDH release after a 24-hour treatment. Data are means ± SEM. One-way ANOVA analysis, followed by Tukey's post-test, was conducted. *** $p < 0.001$ **** $p < 0.0001$ vs control, °°° $p < 0.001$ vs medium M1 100%. Each experiment was repeated three times,

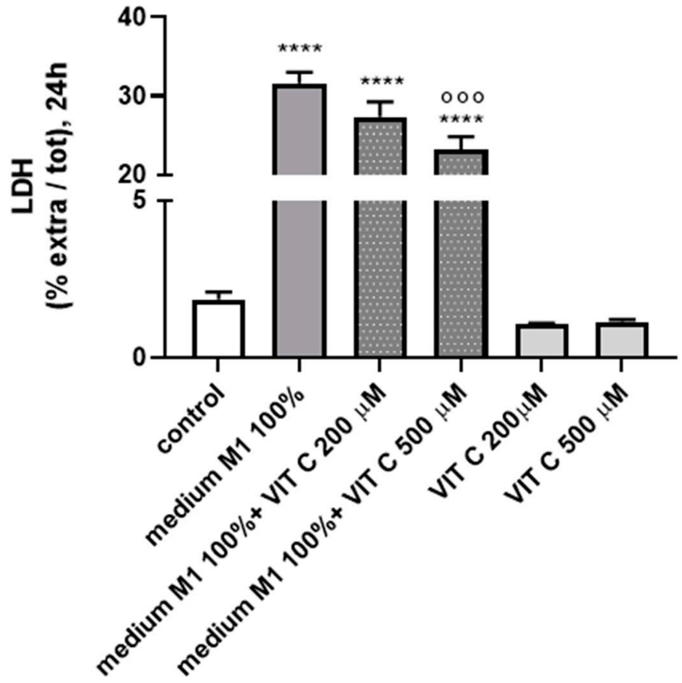

with each experimental group consisting of six replicates.

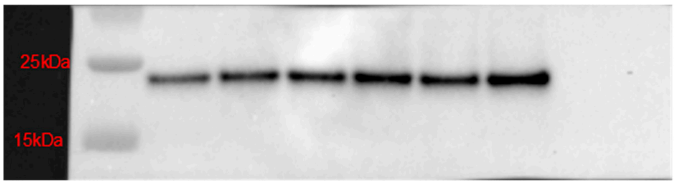

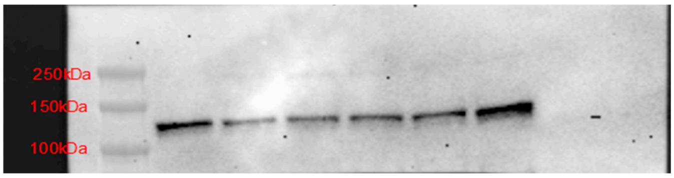

p21

PARP

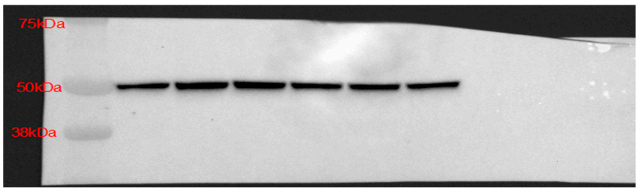

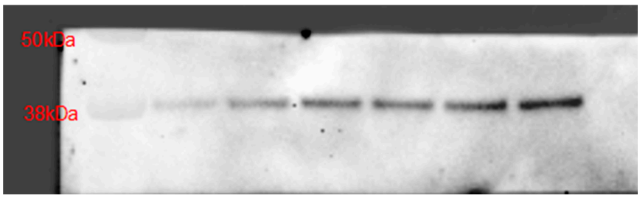

βtubulin

p38

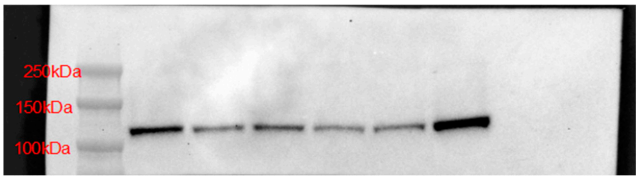

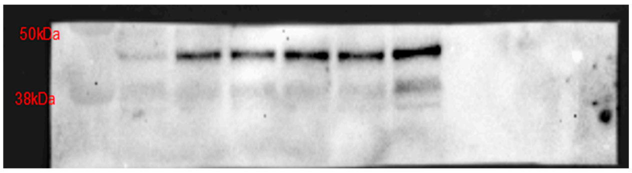

p RB

p MEK

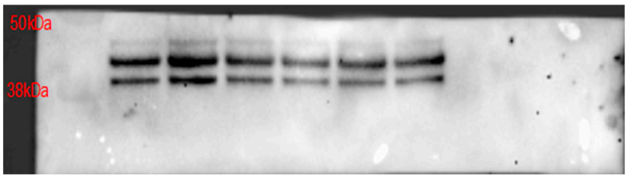

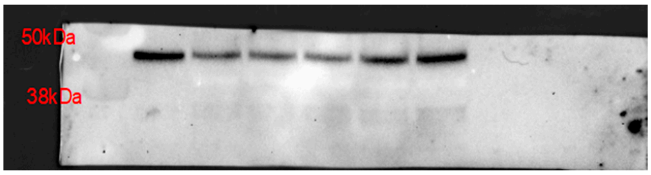

Total ERK

p CREB

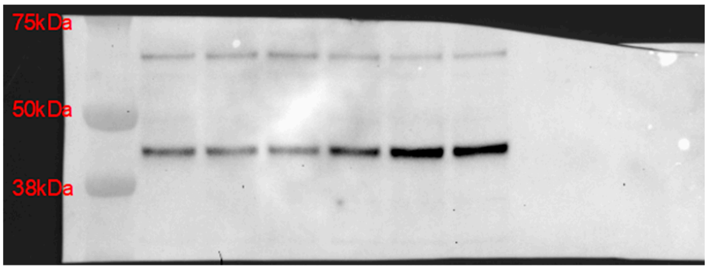

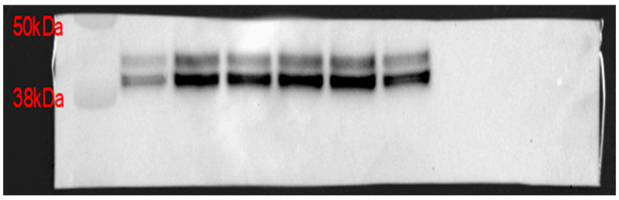

p ERK

Total Creb

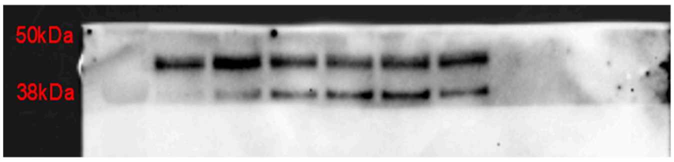

Total MEK

Supplementary Figure S2 . Full-length, uncropped, and unprocessed scans of all Western blots presented in the main manuscript. The lanes are identified as follows: 1: Control; 2: M0 50%; 3: M0 100%; 4: M1 50%; 5: M1 100%; 6: ATR 5 μM.
